# A Review of Human Exposure to Microplastics and Insights Into Microplastics as Obesogens

**DOI:** 10.3389/fendo.2021.724989

**Published:** 2021-08-18

**Authors:** Kurunthachalam Kannan, Krishnamoorthi Vimalkumar

**Affiliations:** Department of Pediatrics and Department of Environmental Medicine, New York University School of Medicine, New York, NY, United States

**Keywords:** microplastics, adipogenesis, PPARs, phthalates, obesogens

## Abstract

The ubiquitous exposure of humans to microplastics (MPs) through inhalation of particles in air and ingestion in dust, water, and diet is well established. Humans are estimated to ingest tens of thousands to millions of MP particles annually, or on the order of several milligrams daily. Available information suggests that inhalation of indoor air and ingestion of drinking water bottled in plastic are the major sources of MP exposure. Little is known on the occurrence of MPs in human diet. Evidence is accumulating that feeding bottles and medical devices can contribute to MP exposure in newborns and infants. Biomonitoring studies of human stool, fetus, and placenta provide direct evidence of MP exposure in infants and children. MPs <20 µm were reported to cross biological membranes. Although plastics were once perceived as inert materials, MP exposure in laboratory animals is linked to various forms of inflammation, immunological response, endocrine disruption, alteration of lipid and energy metabolism, and other disorders. Whereas exposure to MPs itself is a concern, MPs can also be sources of exposure to plastic additives and other toxicants. Exposure of human cell lines to MP additives such as phthalates, bisphenols, and organotins causes adverse effects through the activation of nuclear receptors, peroxisome proliferator-activated receptors (PPARs) α, β, and γ, and retinoid X receptor (RXR), leading to oxidative stress, cytotoxicity, immunotoxicity, thyroid hormone disruption, and altered adipogenesis and energy production. The size, shape, chemical composition, surface charge, and hydrophobicity of MPs influence their toxicity. Maternal transfer of MPs to the developing fetus has been demonstrated in exposed laboratory animals and through the analysis of human placenta. In laboratory animal studies, maternal exposure to MPs altered energy and lipid metabolism in offspring and subsequent generations. Moreover, concomitant with the global increase in plastics production, the prevalence of overweight and obesity in human populations has increased over the past five decades, and there is evidence to support the hypothesis that MPs and their additives are potential obesogens. Even though MP exposures are ubiquitous and toxic effects from such exposures are a concern, systematic studies on this topic remain urgently needed.

## Introduction

**Figure 1:**
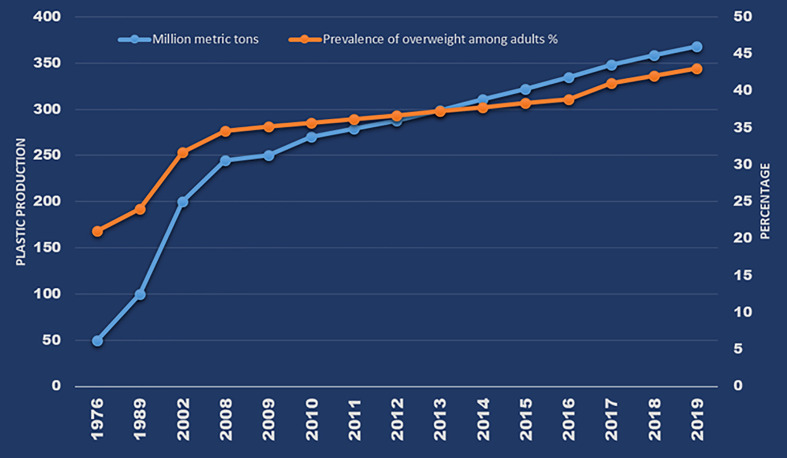
Global annual plastic production and prevalence of overweight over the past 4 decades (Source: Plastics - The Facts 2020, page 16; WHO: https://apps.who.int/gho/data/view.main.GLOBAL2461A?lang=e; Obesity data from: CDC: https://www.cdc.gov/nchs/products/databriefs/db360.htm).

Global plastic pollution is a significant environmental and public health concern (1–5). A total of approximately 8300 million metric tons of plastics were manufactured from 1950 to 2015 (6), and production rate has been increasing, reaching 368 million metric tons (annually) in 2019 (**Figure 1**). Plastics are resistant to chemical and biological degradation and therefore durable. Furthermore, their low density and expense coupled with corrosion resistance make them suitable for use in an enormous variety of consumer products. There are currently at least 45 different types of plastics in commercial use, including polypropylene (PP), polyethylene (PE), polyethylene terephthalate (PET), polystyrene (PS), polyurethane (PU), polyvinyl chloride (PVC), and polycarbonate (PC) (7). According to Plastic Europe (2013) (www.plasticseurope.org/application/files/7815/1689/9295/2013plastics_the_facts_PubOct2013.pdf), of the ~288 million tons of plastics produced in 2012, 85 million tons (~30%) were PE, 54 million tons (19%) were PP, and 31 million tons (11%) were PVC. PE (as low- and high-density material; LDPE/HDPE) is used in producing plastic films (35 million tons annually) that are finished as carrier bags, sandwich bags, food wraps and containers, straws, toys, mulch, irrigation pipes, packaging foam, cling wrap, and electrical cables, among other products. PP is the second most widely used thermoplastic worldwide, in applications ranging from plastic packaging, plastic parts for machinery and equipment, and medical devices to fibers and textiles. PVC is used in buildings (e.g., window shutters, water pipes, and upholstery), shampoo and cooking-oil bottles, insulation for electric cables, and cling films. PS is used in buildings and in moldable materials, especially for the production of disposable plates and cups, meat trays, egg cartons, carryout containers, and electronic goods. PET, which is related to PE, is used in textile fibers for clothing manufacture (as polyester) and in containers for liquids (e.g., water, juice, and soft drink bottles) and foods (8). PET is an excellent replacement product for glass, and its demand is 14.5 million tons annually (Plastic Europe, 2008: https://www.plasticseurope.org/application/files/2815/1689/9283/2006compelling_fact_PubJan2008.pdf). Consumption rates of plastics vary between countries-according to Plastics Insight (https://www.plasticsinsight.com/world-per-capita-consumption-pe-pp-pvc-resins-2014), per capita plastic consumption levels in the United States and India in 2014 were 95 and 8 kg, respectively — but continue to increase on a global basis. Concurrent with the production and use of plastics, the generation of plastic waste has increased exponentially in the past 4 decades (6).

It is generally believed that large plastic polymers are inert and are not absorbed by the intestinal system (due to their size), and therefore are excreted un-metabolized. However, upon entering the environment and/or in biological systems, plastics break down into small particles through biotic and abiotic weathering and transformation processes, creating massive amounts of smaller plastic particles in the environment, including plastic particles of <5 mm that are termed ‘microplastics’ (MPs) (9). An estimated 75,000–300,000 tons of MPs are released by plastics breakdown annually in the EU alone (10). As plastics continue to break down in the environment, the fraction of MPs in the total mass of plastic waste has been predicted to reach 13% by 2060 and will continue to increase (11). MPs are classified as primary or secondary based on their source of environmental release. Primary MPs are those deliberately manufactured at sizes <5 mm for use in applications such as microbeads in facial cleansers/scrubs, shower gels, and scrubbing pads (dish washing), as well as microfibers from clothing (dish cloths and towels) (12–17). Secondary MPs are those generated from plastic polymers through normal weathering processes, including erosion, abrasion, corrosion, photo-oxidation (chemical), and biological transformation (18–21). MPs are also classified based on their physicochemical properties as microfibers from cloth (fleece, diapers, cigarette butts), fragments (larger particles from cutlery and lids), nurdles (plastic pellets), foams (cups, plates, food packaging containers), microbeads (plastics <1 mm in diameter; used in toiletries, facial cleansers, exfoliating soap products and toothpaste), and nanoplastics (plastics 1–100 nm in diameter; potentially derived from disintegration of MPs) (22, 23).

MPs persist in the environment for hundreds to thousands of years. For example, plastic PET water bottles, disposable diapers, and PS foams have been estimated to have lifespans of 450, 500, and >5,000 years in the environment, respectively (https://www.goecopure.com/lifespan-of-plastic.aspx). Not only is pollution by MPs themselves a significant environmental concern, but plastic particles also can release additives and other performance-enhancing chemicals that are incorporated into the polymers during production. These include inorganic fillers (to fortify the plastic), heat stabilizers (to impart heat resistance), plasticizers (to make plastic flexible and pliant), flame retardants (to provide ignition and fire resistance), UV stabilizers (to prevent degradation from sunlight/photo-oxidation), colorants (to provide opacity and matte finish), and luster additives (to enhance appearance). Owing to their high costs, these additives are typically added in relatively small concentrations (8, 24–26), but they may have potent biological effects. Additives including bisphenols, phthalates, benzothiazoles, organotins, and polybrominated diphenyl ethers (PBDEs) leach out of plastics during usage and/or after disposal (8, 27–32). Aquatic and terrestrial pollution by plastics additives has been extensively documented. Furthermore, MPs can act as vectors for various other contaminants, as they sequester organic and inorganic pollutants from the surrounding environment (33).

Several studies have reported the ubiquitous occurrence of MPs in various environmental matrices, including surface water, sediment, wastewater, Arctic and Antarctic sea ice, indoor and outdoor air, bottled water, and select food products (7, 34–44). Whereas studies of MP pollution in the aquatic environment, especially the oceanic environment, have received considerable attention for over a decade, effects of MPs on human health have attracted notice quite recently, following the detection of MPs in seafood, honey, milk, beer, table salt, drinking water, and air (45). Furthermore, the identification of MPs in human placenta raised serious concern about exposure in utero (46). A meta-analysis of existing data on the concentrations of MPs in various sources of human exposure suggested a mean ingestion dose in the range of 0.10–5 g/week (47). Zhang et al. (48) assessed the mass of PET and PC MPs recovered from the feces of dogs and cats, which were used as surrogates for biomonitoring of MPs in humans, and found an excretion rate of 0.03–677 mg MPs/week. These exposure dose estimates are several orders of magnitude higher than those for other toxicants, such as phthalates, bisphenols, and toxic metals. Thus, there is clear evidence of substantial human exposure to MPs.

Although plastics were thought of as inert materials, several studies in recent years have described health risks from exposure to MPs in humans. Among the various toxic effects reported in laboratory animals from exposure to MPs and plastic additives, the disruption of adipogenesis and lipid metabolism through the activation of peroxisome proliferator-activated receptors (PPARs) suggests that MP exposure may be linked to the increasing prevalence of obesity globally. The increase in global plastics production over the past 50 years is in line with the rate of increase in overweight and obesity in human populations (**Figure 1**). Thus, there is an immediate need to assess the sources, pathways, and potential health effects of MPs in humans, with a focus on obesogenic mechanisms, among others. In this review, we summarize available information on the sources and pathways of human exposure to MPs and on their toxic effects, especially those related to obesogenic mechanisms of MP toxicity.

## Sources of Human Exposure to Microplastics

A major difficulty in determining the risks of MPs to human health is the lack of accurate information on exposure doses, which is primarily due to the fact that methods for quantitative determination of MPs in air, water, food, and cosmetics are still evolving. A complicating factor is that the available spectroscopy-based methods report MPs (items, fibers, or particles) in terms of number, size, and shape, whereas in exposure science, doses are most often reported in mass. Reported concentrations of MPs in various sources of human exposure (air, indoor dust, drinking water, beverages, honey, salt, sugar, and other dietary sources) from a number of recent papers are compiled in **Table 1**. Contaminated food and water were thought to be major sources of MP exposure. One study reported a per capita intake of MPs through ingestion of food, water, and dust and inhalation of air of 74,000–121,000 items annually (45). Another study estimated an annual per capita MP intake of 39,000–52,000 items, including 37–1000 from sea salt, 4000 from tap water, and 11,000 from shellfish (42). A probabilistic lifetime exposure model predicted a MP intake rate of 184 ng/capita/d for children and 583 ng/capita/d for adults, through nine different exposure sources (109). Mass (or weight)-based estimates of annual MP ingestion were reported to be 15–287 g/person (47). Through analysis of the feces of cats and dogs, as surrogates for humans, Zhang et al. (48) reported an MP intake estimate of 0.03–677 mg/week for pet animals. Overall, through the analysis of pet feces, daily exposure doses on the order of several tens of milligrams were reported. The contribution of various sources to the total MP exposure dose is delineated below.

**Table 1:** Reported concentrations of microplastics in air, dust, drinking water, sea food, food, beverages and human samples.

Sample type	Location	Polymer type	Size	Concentration	Reference
**Air**					
Indoor	Aarhus, Denmark	Polyester, PE, nylon	0.004-0.398 mm	1.7-16.2 particles/m^3^	(43)
	Edinburgh, UK	PET, PU	<5 mm	1666-1671 particles/m^2/^d	(49)
	Paris, France	Not reported	0.005-0.6 mm	1586 – 11,130 particles/m^2/^d	(50)
Outdoor	USA	Cotton, polyster, nylon, polyolefin, PTFE, PE	0.004 -3 mm	132 particles/m^2/^d	(51)
	Asaluyeh county, Iran	Not reported	0.002-0.1 mm	72 items/m^3^	(12)
	Hamburg, Germany	PET, ethylvinyl acetate copolymers	<0.063->0.3 mm	136.5 -512 particles/m^2/^d	(52)
	Pyrenees mountains, France	PS, PE, PP, PVC, PET	<0.025-2.6 mm	366 particles/m^2/^d	(53)
	Dongguan, China	PE, PP, PS, cellulose	<0.2-4.2 mm	175-313 particles/m^2/^d	(54)
	Yantai, China	PS, PE, PP, PVC, PET	0.005-1 mm	0-602 particles/m^2/^d	(55)
	Paris, France	Not reported	0.005-0.6 mm	2-355 particles/m^2/^d	(56)
	Bushehr port, Iran	PET, PE, nylon, PS, PP	<2.5 μm	5.2 items/m^3^	(57)
**Dust particles**					
Indoor	Several countries	PET	<2 mm	38–120,000 μg/g	(58)
	Shanghai, China	PS, polyamide, PP	50−2000 μm	4.4 ×10^3^ MPs/m^2/^d (mean)	(59)
	Surabaya, Indonesia		3000-3500 μm	212 particles (mean)	(60)
	Tiajin, China	PET	50 μm-2 mm	1550-120,000 mg/kg	(61)
		PC	50 μm-2 mm	4.6 mg/kg	
Outdoor	Tiajin, China	PET	50 μm-2 mm	212-9020 mg/kg	
		PC	50 μm-2 mm	2 mg/kg	
**Drinking water**					
Packed water bottle	Bangkok, Thailand	PET, PE, PP, polyamide, PVC	≥50μm	140 MPs/L	(62)
	Catania, Italy	PET	0.5 – 10 µm	657 ± 633 µg/L	(63)
Glass bottles	Erlangen, Germany	PE	>5 µm	6292 ± 10521 particles/L	(64)
Single use PET bottles				2649 ± 2857 particle/L	
Reusable PETbottles				195047 ± 330810 pigmented particle/L	
Reusable PET bottles				23594 ± 25518 pigmented particle/L	
Bottled water	NewYork, USA	PP, nylon	>100 µm	0-14 MPs/L	(65)
Packed mineral water	Germany	PET, PE, PP, polyamide	50-500µm	28 -241 MPs/L	(66)
Mineral water		PET		1 (only one found) MPs/L	(67)
Drinking water fountain	Metro-station, Mexico city	poly-trimethylene terephthalate	0.1-5 mm	5 ± 2 to 91 ± 14 MPs/L	(68)
Tap water	Qingdao, China	PE, PS, PET, rayon, polyester, polyacrylic, polymethylpentene, polyimide	10 to 5000 µm	0.3 - 1.6 MPs/L	(69)
	North-western Germany	PS, PVC, polyamide, epoxy resin, PE	>20 μm	0-0.0007 MPs/L	(70)
	Minneapolis, Minnesota, USA	Synthetic polymers	0.1-5 mm	5.45 particles/L	(71)
	Czech Republic	PET, PP, PE	<10 μm	469.6 MPs/L (mean)	(72)
	Different parts of China	PE, PP, PET	3 to 4453 µm	0 - 1247 MPs/L	(73)
**Diet**					
Sea salt	Bulgaria	PP	100-5000 μm	12 items/kg	(74)
Rock salt		PP, PE		8 items/kg	
Sea salt	China		45-4300 μm	550-680 items/kg	
Rock salt				43-364 items/kg	
Lake salt				7-204 items/kg	
Sea salt	France	PS	160-980 μm	0-2 items/kg	
Rock salt	Germany		100 μm	2 items/kg	
Rock salt	Hungary	Low density PE	100-4000 μm	12 items/kg	
Sea salt	India		1000-5000 μm	(30-370 items/kg	
Sea salt	Indonesia	PP		1400 items/kg	
Sea salt	Italy		1000-5000 μm	4-30 items/g	
Rock salt				80 items/kg	
Sea salt	Korea	PE	100-3000 μm	100-230 items/kg	
Rock salt	Philippines		100-5000 μm	120 items/kg	
Sea salt	Senegal		100-3000 μm	48 items/kg	
Rock salt				800 items/kg	
Sea salt	Thailand		100-5000 μm	70-400 items/kg	
Sea Salt	USA			50-800 items/kg	
Rock salt				113-367 items/kg	
Sea salt	UK		100-2000 μm	140 items/kg	
Sea salt	Vietnam		100-5000 μm	76-88 items/kg	
Beer	Germany		Not specified	2-79 fibers/L	(75)
				12-109 fragments/L	
				2-66 granules/L	
	Duluth, Minnesota, USA		100-5000 µm	0-14.3 particles/L	(71)
Honey	Germany, France, Italy, Spain and Mexico		10-20 µm	166 ± 147 fibers/kg	(76)
				9 ± 9 fragments/kg	
Sugar				217 ± 123 fibers/kg	
				32 ± 7 fragments/kg	
Honey	Switzerland		500 µm	1760 – 8680/kg (black particles)	(77)
				132 – 728/kg (white fibers)	
				60-172/kg (white particles)	
				32-108/kg (coloured fibers)	
Canned sardines and sprats	Australia and Malaysia		190-3800 µm	20 (mean) items/g	(78)
Seaweed nori	China		100-500 µm	0.9 - 3.0 items/g	(79)
Tea bags	Canada (billion microplastics and 3.1 billion nanoplastics single cup of the beverage)	fibers	25 µm	11.6 items/g	(80)
**Oyster, bivalves and mussels (Seafood)**					
**Location**	**Species name**	**Tissue**	**Size**	**Concentration**	**Reference**
California, USA	*Crassostrea gigas*	Soft tissue	>500 µm	0.6 particles/g	(81)
Brittany, France	*Crassostrea gigas*		5-25 µm	0.47 particles/g	(82)
Shanghai, China	*Meretrix lusoria*		5-5000 µm	9.22 paticles/individual	(83)
	*Mytilus galloprovincialis*		5-5000 µm	4.33 ± 2.62 particles/individual	
	*Patinopecten yessoensis*		5-5000 µm	57.2 ± 17.3 particles/individual	
Italy	*Mytilus galloprovincialis*	Hepatopancreas and gills	760-6000 µm	6.2-7.2 particles/g	(84)
Scottish coast	*Mytilus* spp.	Soft tissue	200->2000 µm	3.2 ± 0.52 paticles/individual	(49)
	*Modiolus modiolus*		200->2000 µm	3.5 ± 1.29 paticles/individual	
Musa estuary, Persian Gulf	*Penaeus semisulcatus*	Muscle, skin	<100->1000 µm	7.8 particles/individual	(85)
Persian Gulf, Iran	*Pinctala radiata*	Soft tissue	10-5000 µm	11 particles/individual	(86)
East China Sea	*Mytilus* spp.		1000-5000 µm	3.69 ± 9.16 items/g	(87)
UK coast	*Mytilus edulis*		500 µm	0.7 to 2.9 items/g	(88)
				1.1 to 6.4 items/individual	
South Korea	*Crassostrea gigas, Mytilus edulis, Tapes philippinarum, Patinopecten yessoensis*		300 µm	Mean: 0.15 ± 0.20 n/g species and 0.97 ± 0.74 n/individual	(89)
Belgium coast	*Mytilus edulis*		200-1500 µm	2.6 to 5.1 fibers/10 g	(90)
Fuzhou, China	*Bivalve*		320-1600 µm	0.11-0.12 items/g and 0.59-1.44 items/individual	(91)
Xiamen, China	*Bivalve*		100-4000 µm	0.28-0.30 items/g and 1.26-1.56 items/individual	
**Fishes**					
Saudi Arabian Red sea coast	*Acanthurus gahhm*	Gastrointestinal tract	2700 µm	10 per g (mean)	(92)
	*Epinephelus areolatus*		1800 µm	10 per individual (mean)	
	*Epinephelus chlorostigma*		1900 µm	3 per individual (mean)	
Northeast Persian Gulf	*Alepes djedaba*	muscle	<100-5000 µm	20 per individual (mean)	(93)
Musa estuary, Persian Gulf	*Cynoglossus abbreviatus*	Muscle, gut, gills, liver, skin	<100->1000 µm	11 per individual (mean)	(85)
Mondego estuary, Portugal	*Dicentrarchus labrax*	Gastrointestinal tract	≤1000-5000 µm	40 per individual (mean)	(94)
	*Diplodus vulgaris*			40 per individual (mean)	
Mediterranean Sea, Spain	*Engraulis encrasicolus*	Liver	124-438 µm	10 per individual (mean)	(95)
		Gastrointestinal tract	Not specified	105 per individual (mean)	(96)
Tokyo Bay, Japan	*Engraulis japonicus*		10-500 µm	64 per individual (mean)	(97)
Goiana estuary, Brazil	*Cynoscion acoupa*	Gut	5000 µm	552 per individual (mean)	(98)
Spanish Atlantic	*Merluccius merluccius*	Stomach	380-3100 µm	12 per individual	(99)
Indian coast	*Sardinella longiceps*	Gut	500-3000 µm	10 per individual	(100)
East China Sea	Wild fish species	Gill	24-268 µm	0.77 ± 1.25 items/individual	(101)
	Crustacean spp.	Gastrointestinal	32-4092 µm	0.52 ± 0.90 items/individual	
South China Sea	Deep sea fishes (13 species)	Stomach and intestine	40 – 200 µm	1.96 ± 1.12 items/individual and 1.77 ± 0.73 items/individual	(102)
Fuzhou, China	Wild fishes	Gastrointestinal	440-11000 µm	0.60-0.65 items/g and 1.69-2.29 items/individual	(91)
Xiamen, China			450-7200 µm	0.49-1.26 items/g and 2.39-4.71 items/individual	(90)
Southern Caspian Sea	*Rutilus frisii kutum*	Stomach	<500 µm	11.4 itmes/fish	(103)
Northern Ionian Sea, Greece	*Mytilus galloprovincialis, Sardina pilchardus, Pagellus erythrinus, Mullus barbatus*	Gills, stomach, intestines	0.5-0.1 mm	1.7–2 items/individual and 1.5–1.9 items/individual	(104)
Bay of Bengal, India	*Marine fish (10 species)*	Gastrointestinal	<500 µm	2.2 ± 0.89 items/individual	(105)
Southern Caspian Sea	*Chelon Aurata, Rutilus kutum*	Gut	1.94 mm (mean)	2.29 MPs/fish	(106)
**Human specimens**					
**Sample type**	**Location**	**Polymer type**	**Size**	**Concentration**	**Reference**
Human placenta	Rome, Italy	PP and others	5-10 µm	12 fragments in 4 placentas	(46)
Human feces	Vienna, Austria	PP, PET	50 to 500 μm	20 MPs per 10 g of stool	(107)
					
		
Lung tissue	Sao Paulo, Brazil	PP, PE, PVC, cellulose acetate, polyamide, PS, PU	<5.5 μm particles and 8.1-16.8 μm fibers	Mean: 0.59 MP/g (470 particles per lung)	(108)
Pet feces	Albany, New York, USA	PET	<2.4 mm	Cat: <2,300-340,000 ng/g dw	(48)
				Dog: 7,700-190,000 ng/g dw	
		PC	<2.4 mm	Cat: <32 to 13,000 ng/g dw	
				Dog: <32-26,000 ng/g dw	

PE, polyethylene; PET, polyethylene terephthalate; PC, polycarbonate; PP, polypropylene; PU, polyurethane; PTFE, polytetrafluroethylene; PS, polystyrene; PVC, polyvinylchloride.

### Indoor/Outdoor Air and Dust

Although diet was previously thought to be the major pathway of human exposure to MPs, recent studies on airborne MPs provide evidence that inhaled indoor air may be the dominant source (45, 110). With their small size and low density, MPs can be suspended and transported by air, and airborne MPs are directly inhaled by humans. At an average reported air concentration of 9.8 MPs/m^3^ (45) and an inhalation rate of 15 m^3^/day, annual inhalation exposure averaged 53,700 particles per person in one study. Distribution of MPs in atmospheric fallout or road dust in Paris (France), Tehran (Iran), Dongguan (China), Kusatsu (Japan), Da Nang (Vietnam), and Kathmandu (Nepal) has been documented (54, 111, 112).

The reported units for MPs in air vary depending on the sampling method. A vast majority of studies measure concentrations in atmospheric fallout or deposition in the units of number of fibers, fragments, particles, or items per m^2^ (for fallout) or per m^3^ (for air) per day (**Table 1**). The reported amounts of airborne (deposition/flux) MPs in cities ranged from 2 to 925 particles/m^2^/day, and those in road dust ranged from 3 to 60 items per gram or 0.4 to 33.4 items per m^2^ (110). One study reported that the deposition rate of MPs in central London (England) varied between 575 and 1008 particles/m^2^/day (113). Much higher deposition rates, of up to 11,130 MPs/m^2^/day, were reported in an indoor environment in Paris (France) (1). The study from Paris reported the occurrence of 0.3–15 (mean: 0.9) and 0.4–56.5 (mean: 5.4) particles/m^3^ in outdoor and indoor air, respectively (1), with concentrations of MPs in indoor air averaging approximately 5–10 times higher than those in outdoor air. Furthermore, the flux of MPs in urban air was approximately two-fold higher than that in suburban air in France (1), and lower atmospheric levels of MPs were reported in dry seasons than in rainy seasons (56). A study from Denmark showed that the most common types of MPs found in indoor air were PET (59–92%), PE (5–28%), PP (0.4–10%), and nylon (0-13%) (43). A study from China identified PET and acrylic fibers as the major airborne MPs in indoor air (59).

**Figure 2:**
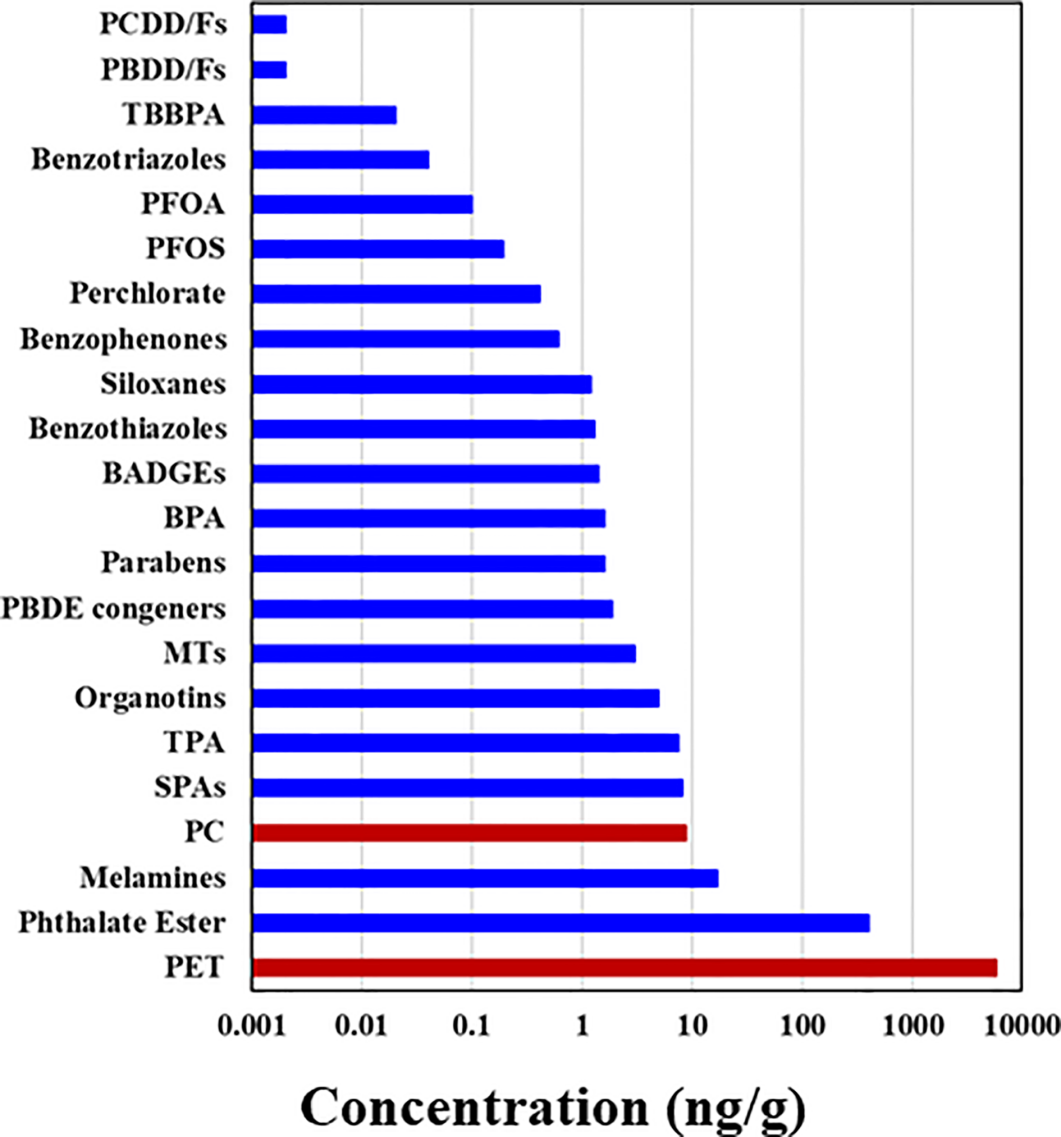
Median concentrations of polyethylene terephthalate (PET) and polycarbonate (PC)-based microplastics measured in indoor dust samples collected from Albany, New York, USA, compared with those of other chemicals [see Zhang et al. (48) for details]; PCDD and PCDFs, polychlorinated dibenzo-p-dioxins and dibenzofurans, respectively; PBDD and PBDFs, polybrominated dibenzop-dioxins and dibenzofurans, respectively; TBBPA, tetrabromobisphenol A; PFOA, perfluorooctanoic acid; PFOS, perfluorooctanesulfonate; BADGEs, bisphenol A diglycidyl ether; PBDEs, polybrominated diphenyl ethers; MT, metabolites of 2,6-di-*tert*-butyl-4-hydroxytoluene; SPAs, synthetic phenolic antioxidants; TPA, terephthalic acid.

The major sources of MPs in indoor and outdoor air were synthetic textiles, degradation of plastics, building materials, waste incineration, and landfills (1, 56). Greater concentrations of MPs found in indoor than in outdoor air were attributed to release from textiles. Indoor air and dust are major contributors to human MP exposure. A study measuring the masses of PET and PC MPs in indoor dust from 12 countries reported the ubiquitous occurrence of PET-based MPs at concentrations of 38–120,000 μg/g (median: 5900 μg/g) and PC-based MPs at <0.11–1700 μg/g (median: 8.8 μg/g) (48). Among various classes of micropollutants measured, PET was the most abundant chemical found in indoor dust thus far (**Figure 2**) (48). Geometric mean exposure doses to MPs through ingestion of indoor dust in adults and children were reported to be on the order of thousands of ng/kg-bw/day, with the highest value (150,000 ng/kg-bw) reported for PET exposure in infants (48). This corresponds to an estimated ~10 mg/day for a person weighing 70 kg. This exposure dose is for PET MP alone, although it may reflect higher end of an exposure dose for that MP, based on the median concentration measured in indoor dust [see Zhang et al. (48) for details]. A few exposure models showed human inhalation doses of MPs in the range of 6.5–8.97 µg/kg-bw/day, with those in infants and toddlers being 3–50 times higher than those in adults (48, 110). Prata et al. (42) estimated average per capita inhalation exposure to MPs ranging between 26 and 130 particles per day. Another study showed that the inhaled amount of airborne MPs reached 272 particles per day (43). In general, a rough estimate of human inhalation and dust ingestion exposure to MPs is on the order of a few milligrams per day. However, inhalation doses of MPs can vary depending on the type of textiles in use and other indoor environmental factors (such as ventilation), and further studies are needed in this area (114).

A human biomonitoring study reported the presence of plastic fibers in pulmonary tissue (115), suggesting that airborne MPs can deposit or accumulate in lungs. Fibrous particles of a few tens of microns in size are reported to deposit in lungs (116). Although alveolar macrophages engulf MPs, particles 15–20 µm in size are toxic (to macrophages) and eventually enter the circulatory and lymphatic systems (117). Other studies have reported that MPs can induce inflammatory response, cytotoxicity, and genotoxicity in lung tissue (118); that occupational exposure to airborne MPs among workers in synthetics mills is associated with respiratory illnesses (42); and that long-term MP exposure leads to lung diseases, including asthma and pneumoconiosis (42, 119). Although a few studies have described the significance of indoor air and dust as the major sources of MP exposure in humans, their relative contributions to cumulative exposure are not known. Further research is needed to quantitatively assess exposure doses to various types and sizes of MPs through air inhalation and dust ingestion, along with the concomitant health effects. Biomonitoring studies of MPs in lung biopsies will increase our understanding of the effects of MPs on respiratory health. Because no standard method exists for determining MP levels in air and dust, efforts are also needed to harmonize the analytical methods as a first step in this direction.

### Drinking Water and Packaged Beverages

MPs have been widely found in freshwater resources, and therefore it is considered that drinking water derived from such sources contain MPs. One study documented lower concentrations of MPs in groundwater compared to tap and bottled water, suggesting that MPs originate from water distribution and/or bottling processes (120). Drinking water treatment can remove up to 88% of MPs through coagulation-flocculation-sedimentation (72). Schymanski et al. (66) reported the ubiquity of MPs in returnable and single-use plastic bottles, beverage cartons, and glass bottles collected in Germany. Most (80%) of the MPs in bottled water were PET and PP of 5–20 µm in size. The average MP contents in drinking water from returnable and single-use containers were 118 and 14 particles/L, respectively. High concentrations of MPs found in bottled water were due to release from the plastic container itself. However, the German study showed that water bottled in glass also had notable levels of MPs (mean: 50 particles/L), and suggested that plastic caps used for glass bottles were the source of MPs.

Reported concentrations of MPs in drinking water vary by several orders of magnitude (**Table 1**) due to the varying analytical approaches used, especially with regard to the use of filter membranes with different pore sizes and different identification methods (e.g., FTIR, Raman, X-ray photoelectron, energy dispersive x-ray spectroscopy, or scanning electron microscopy). This generally precludes direct comparison among studies of MP abundances reported in drinking water. In tap and bottled water, these vary between 0 and 10,000 particles/L (121), with plastic-bottled water containing higher concentrations of MPs (0–10,000 particles/L) than tap water (0–61 particles/L) (65, 71). Reported concentrations of MPs in tap water and bottled water from China, Thailand, Mexico, and Iran were in the range of 0-1247 particles/L, with average concentrations ranging from tens to hundreds of particles/L (**Table 1**).

In an exposure assessment, Cox et al. (45) used mean MP concentrations in bottled and tap water of 94 and 4.2 particles/L, respectively. On the basis of the average water consumption pattern, the mean daily intake of MPs through drinking water was estimated at 48, 55, 47, and 51 MPs by male children, male adults, female children, and female adults, respectively (45). The calculated exposure dose from bottled water was 22-fold higher than that from tap water. It was estimated that an individual drinking exclusively bottled water would ingest 90,000 MPs/year, *vs.* just 4000 particles/year for someone ingesting only tap water. The authors suggested that avoiding the consumption of plastic-bottled water might effectively reduce exposure to MPs (45), given that plastic-bottled water consumption is currently the second-greatest source of exposure to MPs globally.

Besides tap and bottled water, several studies have reported the occurrence of MPs in beverages such as beer, wine, energy drinks, and bottled tea. The concentrations of MPs in beer from Germany, the USA, and Mexico were in the ranges of 10–256, 0–14, and 0–28 particles/L, respectively (122). White wine from Italy contained 2563–5857 MP particles/L (122). Similarly, MP levels of 11–40 particles/L have been reported in soft drinks, energy drinks, and tea from Mexico (122). Another study reported that a single plastic tea bag can release billions (12 billion per cup) of MPs when heated at 95°C (80), making these a significant source of MP exposure. The sources of MP contamination in beverages include source water, packaging material, and the packaging process.

Despite the small number of studies reporting MPs in drinking water, the results vary depending on the analytical methods and experimental protocols, which warrants harmonization of methods. Nevertheless, the ubiquity of MPs in drinking water and beverages is compelling and indicates that drinking water and beverages, especially those packaged in plastic containers, can be the second-largest source of human exposure, after inhalation. Indeed, for individuals consuming only bottled water and beverages packaged in plastic containers, daily MP exposure through this pathway can be higher than that from inhalation (45). More studies are needed to investigate MP concentrations in beverages and other drinks packaged in various types of containers.

### Diet

Among dietary sources, MP contamination of seafood and sea salt has been studied extensively. These studies reported MP concentrations of up to 200,000 particles/kg (123). Among sea salt samples analyzed from various countries, Kim et al. (74) reported that MP concentrations in table salt from Asian countries were higher than those in table salt from other continents. Nevertheless, data from the analysis of MPs in sea salt are prone to discrepancies in analytical approaches, complicating inter-study comparability. Food products reported to contain MPs include bivalves, crustaceans, fish, seaweed, chicken, sugar, salt, and honey (17, 41, 64, 76, 86, 94, 124–126). In 2016, the European Food Safety Authority (EFSA) published an overview of MPs and nanoplastics in food, with a focus on seafood (127). This report highlighted the occurrence of MPs in bivalves, shrimp/crab, and fish at concentration ranges of 0.2–4, 0.75, and 1–7 particles/g, respectively (128, 129). Contamination of honey (40–660 fibers/kg), sugar (217 fibers/kg), and salt (0–19,800 particles/kg) with MPs has been documented (128, 129). Cox et al. (45) used average concentrations in seafood of 1.48 MPs/g, sugar of 0.44 MPs/g, honey of 0.10 MPs/g, and salt of 0.11 MPs/g in a human exposure assessment. Chickens raised in home gardens contained a mean of 63 and 11 MP particles in gizzards and crops, respectively (130). In that study, MPs were identified in chicken feces. In addition, concentrations of MPs increased from soil (0.89 particles/g) to earthworm (15 particles/g) to chicken (130 particles/g). Globally, humans may ingest an average of 0.1–5 g/week of MPs up to 1 mm in size, or 74,000–121,000 particles per year (45). However, the authors of that study noted that this could be an underestimate of actual exposures (45). Major food groups have not yet been analyzed for MP contamination. Furthermore, contamination and release of MPs from plastic food packaging materials are unknown, but could be high, as evidenced by the concentrations reported for plastic-bottled water. One study showed the release of 16 million MP particles/L from PP feeding bottles during sterilization, highlighting the urgent and critical need to assess MP exposure in infants (131). That study showed that exposure in infants fed with milk prepared in PP baby bottles can be as high as 3 million MPs per day. Similarly, steeping plastic tea bags in hot water at 95°C releases 2.3 million MP particles and 14.7 billion nanoparticles into a cup of tea (41, 132). Plastic food packaging could be another important source of MP exposure in humans (133), although little is known on this topic. Kedzierski et al. (134) analyzed the surface of packaged meat products (white chicken breast and turkey) and found PS MPs at concentrations ranging from 4.0 to 18.7 particles per kg of packaged meat. These MPs were found to stick to the meat surface before and after washing. The authors suggested that contamination with plastic particles was due to PS dust suspended in the air of the production facility. Besides food packaged in plastic wraps or containers, carryout fast food packaged in plastics could be a significant source of human exposure to MPs.

Kutralam-Muniasamy et al. (135) analyzed 23 dairy milk samples from Mexico and found an average of 6.5 ± 2.3 MP particles/L (range: 3–11 particles/L), with concentrations being higher in processed milk than in raw milk. The major source of MPs contamination in milk was reported to be sulfone polymers used in filtration in dairy industries (135, 136). The most common types of MPs found in food products were PET, PS, PE, PU, PVC, PP, polyamide, polymethyl methacrylate (PMMA; also known as acrylic or plexiglass), and styrene acrylate (137, 138).

The current understanding is that diet is the third most important source of human exposure to MPs, next to inhalation and drinking water ingestion. However, human diet has not been comprehensively evaluated for MP contamination. Considering the wide use of plastics in food packaging, the potential for dietary contributions to MP exposure is significant, and comprehensive studies are needed to assess MPs in various food products.

### Cosmetics and Textiles

Plastic microbeads (i.e., MP particles generally <1 mm) have been widely used as abrasives in cosmetics, including scrubs and exfoliating soaps, shower gels, sunscreens, shaving foams, shampoos, skin creams, and liquid makeup (139). Hair bleaches, hair colorants, body lotions, lip care products, deodorants, and nail care products also contain microbeads. PE particles have been found in toothpaste and composite dental filling (140). MPs are added to cosmetics for skin exfoliation and cleansing and to deliver opacity, smooth and silky texture, illumination, and viscosity control (Cosmetics Europe 2019: https://cosmeticseurope.eu/news-events/cosmetics-europe-annual-conference-2019). In 2014, 4130 tons of microbeads were used in soap in the EU countries annually (141). Approximately 93% of microbeads used in cosmetics are PE, but they can also be made of PP, PET, PMMA, or nylon (141). Microbead contents in cosmetics vary widely, from 0.05% to 12% (117, 141, 142). On the basis of the amount present in liquid soaps, per capita consumption of MPs in the U.S. population was estimated at 2.4 mg PE particles per day (143). Similarly, facial scrubs collected from the UK contained 1–10 g of PE microbeads per 100 mL, and per capita consumption of PE MPs in facial scrubs was estimated at 0.5–215 mg/day (144). It was estimated that between 4594 and 94,500 microbeads were released into the aquatic environment from a single use of a facial scrub (144).

Given this widespread occurrence of MPs in cosmetics, dermal exposure to MPs cannot be ruled out (145). Many cosmetics are directly applied to the skin, and particles <100 nm can cross the epithelial barrier. MPs used in cosmetics were evaluated by the Federal Institute for Risk Assessment of Germany, which concluded that MPs in these products can be associated with skin damage due to inflammation and cytotoxicity (146). Notably, MPs induce oxidative stress in human dermal epithelial cells.

The release of microfibers during washing of synthetic textiles has also been confirmed as a source of primary MPs in the environment (147). Over 700,000 microfibers were found to be released from a 6-kg wash load of acrylic fabric (147), and up to 13 million microfibers from polyester fabric in the first wash cycle (148). Use of face masks made of synthetic fibers during the Covid-19 pandemic may also have contributed to inhalation exposure to MPs from textiles. To date, no systematic investigations have been conducted to document human MP exposure from the use of cosmetics or synthetic textiles, and further studies are needed in both regards.

## Plastic Additives as Co-Contaminants in MPs

A wide variety of additives and performance-enhancing chemicals (e.g., flame retardants, plasticizers, antioxidants, and light/heat stabilizers) are added to plastic polymers during production. These additives can leach from plastic debris or MPs released into the environment. An estimated 35–917 tons of additives are reported to be released into oceans annually (149), with PBDEs, phthalate esters, nonylphenol, and bisphenol A (BPA) being the commonly known plastic additives (150). The majority of these additives are present in plastic products at concentrations of up to 20% w/w, although some plasticizers can be present at concentrations as high as 70% w/w. Further details about additives have been described in earlier studies (150). It has been reported that over 10,000 chemicals are potentially used as plastic monomers, additives and processing aids, of which over 2400 were identified as substances of concern (151). Brominated flame retardants such as PBDEs, hexabromocyclododecane (HBCD), and tetrabromobisphenol A (TBBPA) have been widely used in plastics to reduce flammability. Owing to health concerns, these flame retardants were replaced by substitutes such as 1,2-bis(2,4,6-tribromophenoxy)ethane (BTBPE), decabromodiphenylethane (DBDPE), and hexabromobenzene (HBB). More recently, organophosphate esters such as tri-*n*-butylphosphate (TnBP) have come into use as flame retardants and as plasticizers (imparting flexibility and malleability) in plastics. Phthalates such as di(2-ethylhexyl) phthalate (DEHP) are the most commonly used plasticizers in PVC plastic; 7.5 million tons of plasticizers are consumed globally every year, with DEHP accounting for 37% of this market (ECPI 2016: https://www.plasticisers.org/plasticisers/). Due to health and regulatory concerns, DEHP has gradually been replaced by diisononyl phthalate (DiNP), diisodecyl phthalate (DiDP), and di(2-propylheptyl) phthalate (DPHP), which collectively represented 57% of plasticizer consumption in Europe in 2015 (ECPI 2016: https://www.plasticisers.org/plasticisers/). The most common plasticizers include esters such as adipates, azelates, citrates, benzoates, *ortho*-phthalates, terephthalates, sebacates, and trimellitates. BPA is a monomer used in the production of PC plastics that is produced at >3 million tons annually. BPA can also be used as an antioxidant or plasticizer in other plastic polymers (PP, PE, and PVC) (152). Other bisphenol analogs, such as bisphenol B, bisphenol F, and bisphenol S, are also used in plastics (153). Nonylphenols (NP) are intermediates of the degradation of nonylphenol ethoxylates (NPE) surfactants and antioxidants (154) and are also used as antioxidants and plasticizers in plastics production (152). Many synthetic polymers also contain antioxidants that are used as additives/stabilizers, including polyolefins (mainly PE and PP), which represent 60% of the global demand for antioxidant additives (https://www.academia.edu/8665160/Polymer_Science). Phosphites are also widely used as antioxidants in plastics, serving to delay oxidation and prevent ageing of plastics (155); (https://www.solvay.com/en/chemical-categories/polymer-additives-uv-light-stabilizers-antioxidants-and-antistatic-agents/brands-chemistries). Benzophenones, benzothiazoles, triazines, benzoxazinones, and benzotriazoles are widely used as UV stabilizers in plastics (156, 157). Similarly, quaternary ammonium salts are used as antistatic agents in plastics. Organotin compounds such as butyl-, phenyl-, methyl-, and octyltins are used as heat and light stabilizers in PVC plastics (158). Human exposures to phthalates (159, 160), bisphenols (153, 161), UV stabilizers such as organotins and benzotriazoles (158, 162), and brominated flame retardants (163) are well known. Further details of human exposures to these chemical additives found in plastics are provided in various publications listed above. It is important to note that exposure to MPs can contribute to and augment exposure to such additive chemicals.

## Uptake of MPs and Occurrence in Human Tissues

The accumulation of plastic particles (PVC) of size 5–110 µm in animal models such as rats, dogs, and pigs was reported as early as 1970 (164). The size of MPs determines their uptake efficiency through gastrointestinal, alveolar, and dermal epithelium (165–167). Once ingested, >90% of MPs were reported to be excreted in feces (168), especially large particles >150 µm; however, smaller particles may be absorbed systematically. It has been reported that MPs 0.1–10 µm in size can cross the blood-brain barrier and the placenta (127), particles <150 µm can cross gastrointestinal epithelium, and particles <2.5 µm can enter the systemic circulation through endocytosis (33). In an engineered drug-delivery system, MPs <5 µm were shown accumulate in the macrophages and be carried to mesenteric lymph nodes, blood circulation, and spleen (169). Controlled exposure studies using human cells and rodents suggest that MPs of <10 µm can also be translocated from the gut to the circulatory system and accumulate in liver, kidney, and brain (170). The smallest particles (<0.1 µm; i.e., nanoplastics) can cross cell membranes, the placenta, and the blood-brain barrier (171). There is evidence for maternal transfer of MPs in exposed laboratory animals: Fournier et al. (128) demonstrated the translocation of 20-nm PS MPs from maternal lungs to fetus in exposed rats, and rats exposed to PS MPs *via* intratracheal instillation during gestation showed accumulation in maternal lungs, heart, placenta, and spleen and in the fetal liver, lungs, heart, kidney, and brain. Wick et al. (172) showed that PS MPs 240 nm in size can cross the placental barrier through diffusion or binding to cellular transport proteins. Although the intestinal absorption rate of MPs is low (<1%), better insight into the uptake of MPs of various sizes, shapes, doses, and types is needed to understand the risks of MPs in humans. Similarly, little is known about the uptake and translocation of MPs through inhalation, although particles <10 µm have been found to be absorbed through alveolar epithelium. The molecular weight cut-off for biliary excretion of anionic chemicals has been reported as 500 ± 50 Da for humans (173, 174). In general, larger MPs are likely excreted through feces or through mucociliary clearance after deposition in lungs. Size, shape, dose, surface functionalization, and charge, as well as hydrophobicity, can affect the uptake, translocation, and accumulation of MPs (170). The toxicity of MPs increases with decrease in size (167).

Accumulation of plastic fibers in lungs was first reported in 1998, with 87% of 114 malignant and non-neoplastic lung specimens found to contain MPs (115). This suggested that MPs can accumulate in lung tissues and could contribute to adverse health outcomes, including cancer. Like 2.5-µm airborne particulate matter (PM_2.5_), MPs should be considered in future monitoring studies and exposure assessments. Earlier studies had reported the occurrence of plastic particles originating from prosthetic medical devices in tissues from patients; PE wear particles of up to 50 µm were found in the liver, spleen, and abdominal lymph nodes of patients with hip or knee replacements (175). Studies have also reported the occurrence of MPs in human and pet animal stool specimens (48, 107). An analysis of stool from eight human volunteers (3 men and 5 women aged 33–65 years) from several countries for MPs 50–500 µm in size found an average of 20 pieces of MPs per 10 g of stool, with PP (62.8%) and PE (17%) being most widely identified. The average excretion rate of MPs in human stool was estimated at 25 particles per 100 g of stool (107). Similarly, the mass of PET and PC MPs was assessed in stools from pet dogs and cats with an excretion rate of calculated in the range of 0.03–677 mg/week (48).

Biomonitoring studies of human body burdens of MPs are still in their infancy. A recent study that reported the occurrence of MPs in human placenta received considerable attention (46), given that they could affect the developing fetus. Ragusa et al. (46) analyzed six human placentas from Rome (Italy) by Raman microspectroscopy and reported 12 MP fragments (5–10 µm) in four of them. All of the MPs were pigmented, and three were identified as PP. The authors hypothesized that MPs in placenta could affect major cellular pathways involved in the immune system, growth factor signaling, and several other systems. A recent study from Malaysia reported the occurrence of MPs in colon at concentrations of 331 particles per specimen or 28 ± 15 particles per g colon, with PC being most abundant, followed by polyamide and PP (176).

Overall, there is convincing evidence that exposure to and accumulation of MPs in human tissues is prevalent. Particles <100 µm can cross cell membranes of exposed tissues/cells/laboratory animals, and particles <20 µm may be efficiently translocated into various organs. Although research in this area is at its infancy, the available evidence points to an urgent need for studies on the uptake, accumulation and health effects of MP exposure in humans.

## Toxic Mechanisms of Microplastics in Relation to Obesity

Little is known about the potential toxic effects of MPs in humans. However, it is reported that when inhaled or ingested, MPs <20 µm in size can penetrate biological membranes, accumulate in tissues, and elicit cytotoxic and immune responses. Exposure of laboratory animals or cell cultures to MPs results in inflammation, cytotoxicity (e.g., oxidative stress, cells injury, cell viability, altered membrane function; 177, 178), genotoxicity (through oxidative damage; 179), and immunotoxicity (180) at the cellular level. MPs can be engulfed by endocytes, and thus reach the cytosol and interact with organelles such as mitochondria and nucleus, causing disruption of cellular processes. Most studies indicate that these responses to MPs are mediated by oxidative stress. For example, Deng et al. (181) reported oxidative stress in mouse liver following exposure to PS MPs. Oxidative stress could aggravate abnormal lipid metabolism, among various adverse health outcomes (**Table 2**). Several of the observed toxic effects of MPs are intricately interconnected, such that disturbance of one process may initiate a cascade of other toxicological responses. Although there is still a paucity of data on the health risks of MPs in humans, there is sufficient evidence to necessitate a precautionary approach in dealing with such exposures. It is also worth to note that the European Commission’s Science Advice for Policy (SAPEA 2016: https://www.sapea.info/wp-content/uploads/report.pdf) and the World Health Organization (WHO 2019: https://www.who.int/publications/i/item/9789241516198) reported that currently no evidence exists for direct adverse effects of MPs on human health. Furthermore, few available observations were mostly based on *in vitro* and laboratory animal studies. A few review articles have reported toxic effects of MPs; here, we focus specifically on the potential the toxic effects related to obesogenic mechanisms.

**Table 2:** Toxic effects of microplastics as related to obesogenic effects in laboratory animals (mostly mice).

Type of microplastics and dosage	Tissue accumulation and uptake	Toxic effect	References
PS microspheres 5 and 20 µm, 0.01-0.5 mg/day	Accumulation in gut, liver and kidney	Changes in lipid profile and improper energy metabolism (reduction in ATP levels), and fatty liver	(181)
PS particle (0.5 and 50 µm)		Decreased body, liver and lipid weight, altered gut microbiota, and changes in lipid metabolism	(182)
PS and PE (0.5-1.0 µm)	PS and PE beads found in gut and liver	Metabolic disorder	(183)
PS (5 µm with doses 100 and 1000 µg/L)	Accumulation in mouse gut part	Gut microbiota dysbiosis, bile acids metabolism disorder	(184)
PS (5 and 20 µm)	Accumulation in gut, liver and kidney	Changes in ATP synthesis and lipid metabolism	(185)
PS (5 µm)		Altered serum and liver markers, changes of metabolic disorder in the gut and glycolipid metabolism, maternal exposure caused metabolic effects in F1 and F2 generations showing transgeneration effects	(186)
PS (0.5 and 5 µm)		Changes in serum and liver metabolic markers and maternal exposure caused fatty acid metabolic disorder in the F1 offspring	(186)
PS (10-150 µm)		Affect the diversity of gut microbiota	(187)

**Figure 3:**
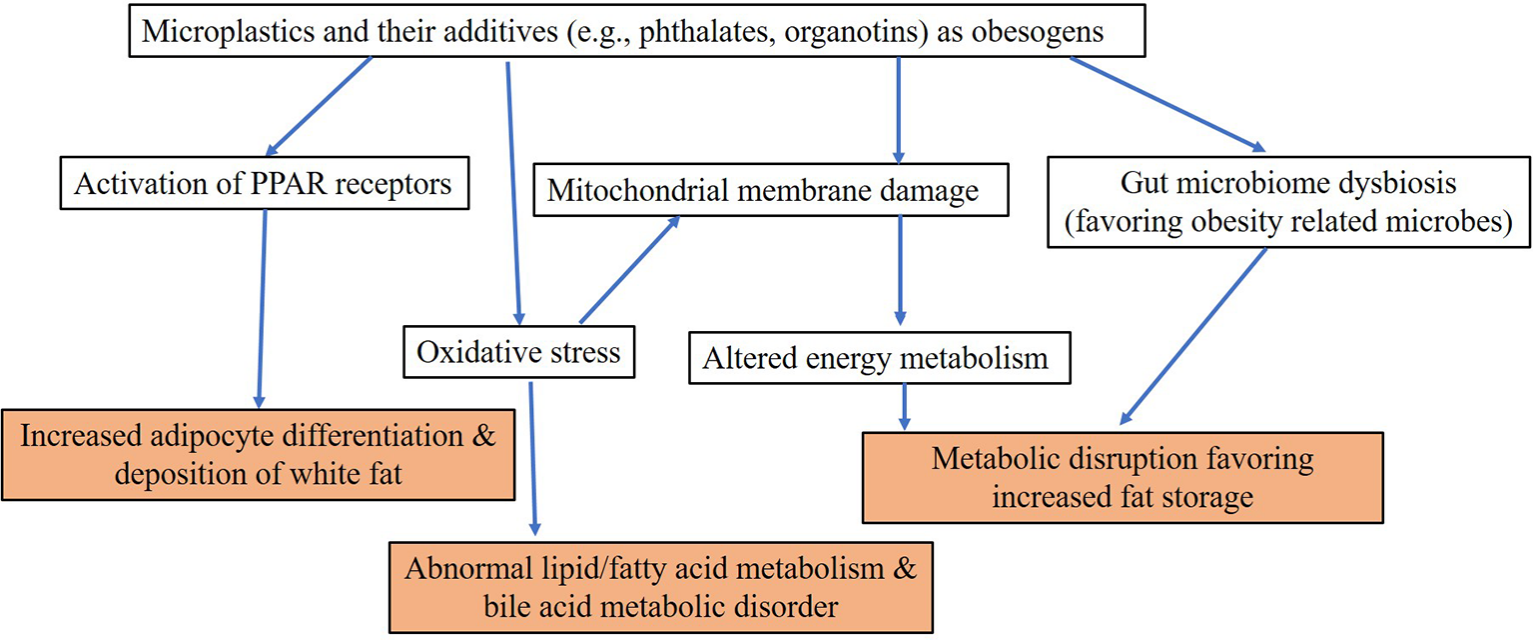
Proposed mechanisms of obesogenic effects of microplastics and their additives.

It is suggestive that over the past five decades, the prevalence of obesity/overweight has increased by three-fold worldwide, which is in congruence with the use of plastics (**Figure 1**). According to the WHO, 39% of adults worldwide were overweight and 13% were obese among those aged ≥18 years in 2016 (http://www.who.int/news-room/fact-sheets/detail/obesity-and-overweight). Overweight and obesity are also on the rise among children, with approximately 30% of U.S. children grouped into those categories in 2013. Although this has been attributed to factors such as excessive caloric intake, inadequate physical activity, and sedentary lifestyle, exposure to environmental contaminants is also proposed to play an important role (188, 189). Data from the National Health and Nutrition Examination Survey (NHANES) showed that caloric intake and energy expenditure were similar in US adults between 1988 and 2006, although there was a 2.3 kg/m^2^ increase in BMI between the two periods (190). Thus, diet alone cannot explain the increase in BMI in recent years. We hypothesize that the global obesity pandemic is associated with exposure to obesogens including MPs and plastic additives. Obesogens are defined as chemicals that lead to increased white adipose tissue accumulation, *in vivo*, after exposure (191). Obesogens affect the differentiation of white adipocytes (192). MPs have been shown to affect adipocyte differentiation following accumulation in liver and kidney and alterations in energy balance and lipid metabolism (181). A few studies that reported obesogenic effects of MPs are listed in **Table 2**. The majority were focused on PS MPs, with little known about the effects of PP, PET, and other types of MPs—topics that also merit investigation. Deng et al. (181) reported decreases in triglyceride and total cholesterol levels in mouse liver following exposure to 5 and 20 µm PS MPs, and confirmed that MPs induced lipid metabolism imbalance and metabolic alterations, including decreases in ATP production and lipid metabolism (181). Changes in liver lipid profiles and lipid metabolism following exposure to PS MPs have been documented in several other *in vivo* mouse exposure studies and *in vitro* human cell bioassays (166, 182, 185, 186, 193). Exposure of mice to PS MPs decreased mRNA levels of key genes involved in lipogenesis and triglyceride synthesis in mouse liver and epididymal fat (182). Jin et al. (184) reported alterations in transcriptional levels of CYP7a1 and ABCb11, two proteins involved in bile acid synthesis and transport in the livers of mice exposed to 5-µm PS MPs. Bile acid plays an important role in lipid metabolism. Maternal exposure to PS MPs during gestation can cause metabolic disorders in the offspring (166), which suggested epigenetic changes and transgenerational effects from MP exposure. These results have considerable implications in regard to early life MP exposure and metabolic alterations and obesity in humans. Other metabolic disorders reported following exposure to MPs in laboratory animals include those affecting energy and bile acid metabolism, as well as alterations in gut microbiota (128, 181, 185). Gut microbiota dysbiosis is a common effect of MPs found in several laboratory mouse studies (128, 166, 182, 187). Changes in gut microbiota can perturb physiological homeostasis, leading to diseases in other organs such as kidney disorders (194), cardiovascular system disorders (195), inflammation and cancer (196), and neurological disorders (197). Various molecular mechanisms leading to obesogenic effects of MPs and plastic additives are outlined in **Figure 3**. Further studies are needed to confirm obesogenic effects of MPs and plastic additives.

In addition to the particle toxicity induced by MPs, the indirect toxicity caused by the release of additives and monomers is of considerable importance. One of the most well characterized obesogens is organotins, which are used as heat and light stabilizers in PVC (198). Butyltins bind to PPARγ and RXR at nM concentrations, promoting adipogensis and lipid accumulation (191). Plastic additives such as phthalates and bisphenols have been reported to affect adipogenesis and energy balance (199). Monoethylhexyl phthalate (MEHP) and diethylhexyl phthalate (DEHP), induced adipogenesis *via* activation of PPARγ (200). BPA, another plastic additive, may bind to estrogen receptors and interfere with estrogen signaling to elicit obesogenic effects (201). Similarly, UV filters such as benzotriazoles (202), flame retardants such as HBCD (203) and BPA (204) have been shown to affect adipogenesis in laboratory animals. Obesogens are believed to act through binding to PPAR isoforms that results in a cascade of activities affecting cellular functions. In general, environmental obesogens activate PPARγ and its heterodimeric partner, the retinoid X receptor alpha (RXRα). PPARγ is considered the master regulator of adipogenesis: its activation increases the expression of adipogenic genes, promotes adipogenic differentiation and increases lipid accumulation. Phthalates, bisphenols, organotins, and HBCD have been identified as PPAR agonists and contributors to obesogenic effects (205), pointing to potential mechanisms whereby plastic additives released from MPs could contribute to obesity. Recent studies have demonstrated other novel mechanisms of obesogenic action, which include induction of epigenetic modification in fat tissue and induction of gut microbiome dysbiosis (191). It is intriguing to note that some studies have reported trans-generational effects of obesogens including MPs (166, 206). It was shown that obesogenic effects of tributyltin were transgenerational and was detected in the F1, F2, F3, and F4 descendants of F0 mice exposed during pregnancy (207). Thus, trans-generational effects of MPs are a concern and subject of further studies.

## Conclusions

MPs are pervasive in the global environment and have reached every compartment of the human food chain. Human exposure to this class of chemicals is widespread and likely to increase, unless adequate mitigation strategies can be implemented. Evidence suggests that MPs of size <20 µm can penetrate organs, and MPs <10 µm can penetrate cell membranes and cross the placental barrier in exposed cells or laboratory animals. Despite this, little is known about the toxic effects of MPs in humans, which might vary depending on the type, size, shape, concentration, and charge of MPs, among other factors. More research is therefore very much needed to understand the cellular and molecular mechanisms of MP toxicity and associated pathology. MPs and several additives associated with their component plastics are obesogenic compounds and that further research is needed to tease out the mechanisms involved and potential means to mitigate these effects. Because MPs and their additives may have multigenerational or transgenerational effects, further efforts to elucidate their mode of toxicity and mitigation of exposures should be an urgent public health goal.

## Author Contributions

KK - wrote the review, data analysis, and figures. KV - reviewed the document, organized references, and prepared tables. All authors contributed to the article and approved the submitted version.

## Funding

The research reported in this manuscript was supported in part by the National Institute of Environmental Health Sciences (NIEHS) under Award Number U2CES026542.

## Author Disclaimer

The content is solely the responsibility of the authors and does not necessarily represent the official views of the NIH. The use of trade names does not imply endorsement of the product by the authors or affiliated institution or the funding agency.

## Conflict of Interest

The authors declare that the research was conducted in the absence of any commercial or financial relationships that could be construed as a potential conflict of interest.

## Publisher’s Note

All claims expressed in this article are solely those of the authors and do not necessarily represent those of their affiliated organizations, or those of the publisher, the editors and the reviewers. Any product that may be evaluated in this article, or claim that may be made by its manufacturer, is not guaranteed or endorsed by the publisher.
